# Inhibition of PfCLK3 interferes with malaria parasite RNA splicing and explains the mechanism of action of a new class of antimalarial candidates

**DOI:** 10.1128/aac.01561-25

**Published:** 2026-06-22

**Authors:** Omar Janha, Niniola Olaniyan, Go Ka Diam, Saumya Sharma, Dario Beraldi, Andrew G. Jamieson, Zbynek Bozdech, Andrew B. Tobin, Katarzyna Modrzynska

**Affiliations:** 1 Keltic Pharma Therapeutics Limited, Advanced Research Centre, University of Glasgow 3526, Glasgow, United Kingdom; 2 School of Molecular Biosciences, Advanced Research Centre, University of Glasgow 3526, Glasgow, United Kingdom; 3 School of Biological Sciences, Nanyang Technological University 54761, Singapore, Singapore; 4 School of infection and Immunity, College of Medical, Veterinary and Life Science, University of Glasgow 3526, Glasgow, United Kingdom; 5 School of Chemistry Advanced Research Centre, University of Glasgow 3526, Glasgow, United Kingdom; The Children's Hospital of Philadelphia, Philadelphia, Pennsylvania, USA

**Keywords:** TCMDC-135051, target validation, PfCLK3, drug discovery, malaria, splicing, protein kinases, mitogen-activated protein kinases, mechanisms of action

## Abstract

Emerging resistance to front-line antimalarials means there is an urgent need to discover new drugs with novel mechanisms of action that remain effective across multiple stages of the parasite life cycle. Previously, we reported a malaria parasite protein kinase, *Pf*CLK3, as a target offering a cure, prophylaxis, and transmission blocking. The homology between *Pf*CLK3 and human kinases suggested that the mechanism of parasiticidal activity of *Pf*CLK3 inhibitors is disruption of RNA processing. Here, we use RNA sequencing to reveal that selective *Pf*CLK3 inhibition extensively affects RNA splicing with 2,039 splice-junctions across 1,125 genes mis-spliced in treated wild-type parasites compared to controls. The function of mis-spliced transcripts showed that the affected genes were involved in numerous essential parasite processes associated with multiple life cycle stages and revealed transcripts and introns particularly susceptible to inhibition. Our study supports the role of *Pf*CLK3 as an important regulator of spliceosome activity and establishes the distinct mechanism of parasiticidal activity of *Pf*CLK3 inhibitors from current front-line treatments.

## INTRODUCTION

Despite significant gains in the fight against malaria, progress in the last two decades has stalled, partly due to the emergence and spread of parasites resistant to the front-line antimalarial drug artemisinin and its partner drugs in artemisinin combination therapies (ACTs). Hence, new antimalarial drugs with alternative modes of action against novel targets are critically needed (1). Ideally, these novel targets should be conserved across parasite species, especially those bearing the highest impact, *P. falciparum* (causing most cases and deaths annually) and *P. vivax* (1). Among the 36 eukaryotic protein kinases reported as essential for the survival of the asexual blood stage of the most virulent human malaria parasite species, *P. falciparum* (2, 3), the cyclin-dependent like protein kinase family (CLK family) made up of 4 members (*Pf*CLK1-4), have emerged as potential drug targets (2, 4). Prominent among this kinase family is *Pf*CLK3, which is established as essential for parasite growth (2, 3, 5) and is highly conserved among closely related human malaria *Plasmodium* species (*P. falciparum*, *P. knowlesi*, and *P. vivax*) as well as the rodent malaria model, *P. berghei*. In this way, it is suggested that the development of drugs inhibiting *Pf*CLK3 would have the potential to offer a cure, prophylaxis, and transmission blocking medicine that is active across all human *Plasmodium species* as shown earlier (4, 6).

The similarity of the *Pf*CLK family to mammalian CLKs and to the serine-arginine-rich protein kinases (SRPK) family suggests that this kinase family, including *Pf*CLK3, phosphorylates SR proteins (4, 7, 8). SR (serine/arginine-rich) proteins are key regulators of alternative splicing that bind exonic splicing enhancers (ESEs) through their RNA recognition motifs (RRMs), which, in turn, mediate protein-protein and protein-RNA interactions via their RS repeat-containing domain (8–10). In *in vitro* assays, *Pf*CLK3 was shown to phosphorylate *Plasmodium* SR proteins, specifically *Pf*SRFR12 and *Pf*SFRS4 as substrates (7). The full list of its targets, however, remains unknown. The kinase catalytic domain of *Pf*CLK3 is closely related to the human splicing factor kinase PRP4 kinase (PRPF4) (4) supporting a role for this kinase in RNA processing (11–14). We have previously described TCMDC-135051—a compound selectively inhibiting *Pf*CLK3 in recombinant kinase assays with no detectable activity against the closely related *Pf*CLK1 or other parasite kinases, including *Pf*PKG and *Pf*CDPK1. The same compound also had no detectable activity against human PRP4 kinase *in vitro* and showed no toxicity toward human hepatocytes (HepG2 cells) up to 10 µM concentration (4, 6). Importantly, we previously identified a variant of *Pf*CLK3 (G449P) that is significantly less sensitive to TCMDC-135051 inhibition. A genetically engineered parasite line expressing this variant in place of the wild-type kinase (henceforth referred to G449P) shows approximately 1.5 log-fold reduction in the parasiticidal potency of TCDMC-135051 (4), further validating direct interaction between *Pf*CLK3 and TCMDC-135051.

Initial microarray studies from our laboratory reported that inhibitory concentrations of TCMDC-135051 differentially regulate genes essential for parasite survival (4). This effect encompassed a substantial proportion of the parasite transcriptome and disproportionally affected genes containing multiple exons, further supporting the role of *Pf*CLK3 in splicing. Despite these early studies and an advanced medicinal chemistry program developing inhibitors to *Pf*CLK3 (6, 15–17), there has been no direct investigation of *Pf*CLK3’s role in RNA processing.

Here, we present total RNA sequencing following TCMDC-135051 pulse treatment of wild type (WT) and genetically engineered parasites expressing an inhibitor-resistant variant of *Pf*CLK3, G449P, in a study designed to investigate the impact on RNA processing (4, 18). We confirm that *Pf*CLK3 is an important regulator of spliceosome activity and established the mechanism of parasiticidal activity of *Pf*CLK3 inhibitors as disruption of RNA splicing. This work also provides a framework for further investigation of the mechanism of splicing in *Apicomplexa* and optimisation of CLK3-selective inhibitors.

## RESULTS

**Fig 1:**
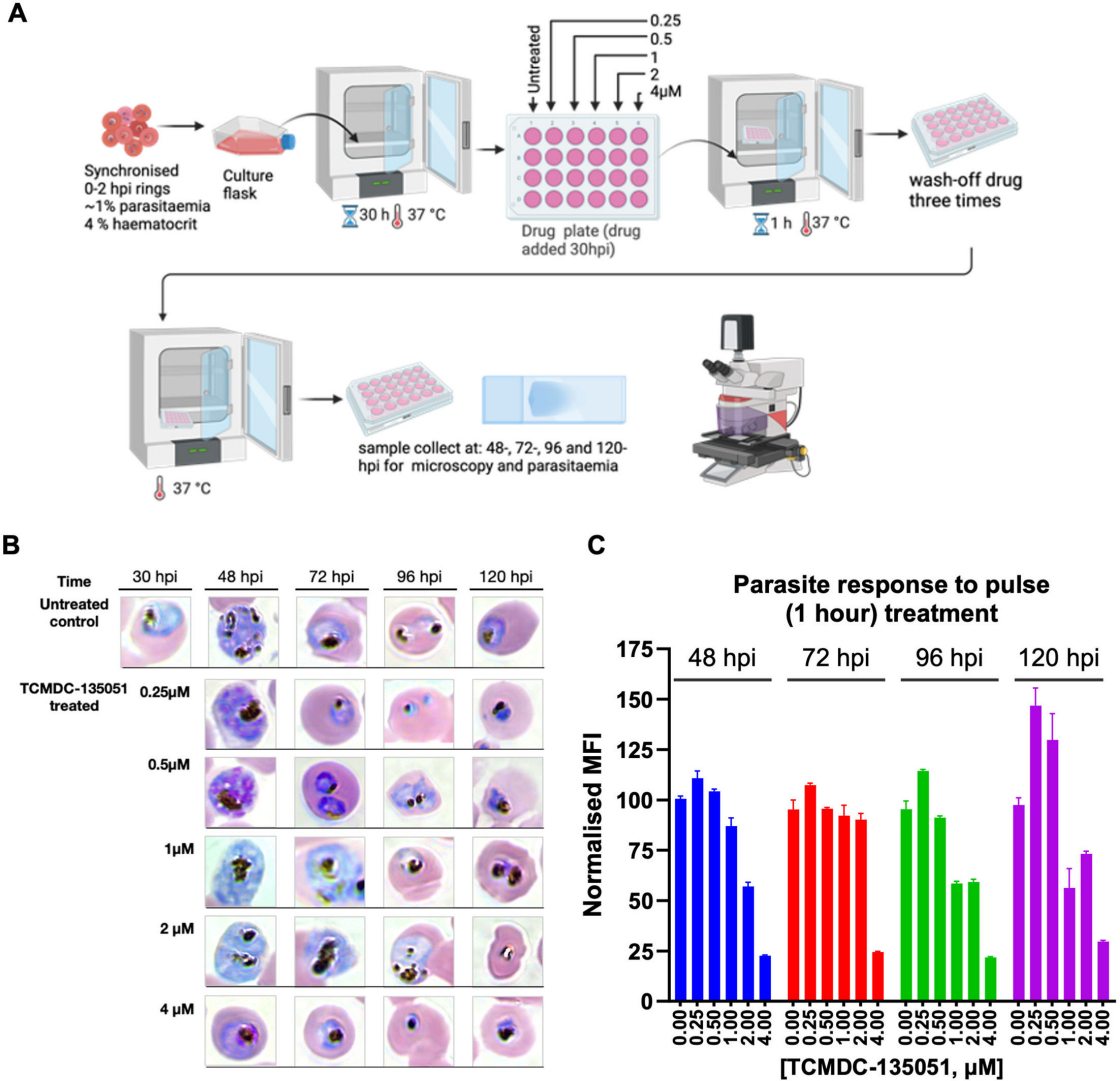
Schematic representation of workflow for pulse treatment, and Giemsa images. (**A**) Step-by-step workflow for culturing synchronized parasites to the desired treatment time of approximately 30 h post-invasion (30 hpi). The parasites were then exposed to various drug concentrations and incubated for 1-h at 37°C before been washed thrice to remove drug pressure. Samples were taken at 48, 72, 96, and 120 hpi (Created with BioRender.com). (**B**) Images of Giemsa-stained thin smears collected at 48, 72, 96, and 120 hpi. The images are representative of three independent experiments in triplicates. (**C**) Parasitaemia mean fluorescence intensity (MFI) readout following incubation for up to 120 hpi. Error bars are the standard error of three independent experiments performed in triplicate (*N* = 9).

To investigate the role of *Pf*CLK3 in splicing, we first established conditions under which the *Pf*CLK3 inhibitor TCMDC-135051 could inhibit *Pf*CLK3 activity without inducing parasite death, thereby avoiding extensive secondary transcriptomic changes. We had previously determined that the EC₅₀ of TCMDC-135051 for parasite killing was approximately 200 nM in a 72-h drug assay (4). Using this as a benchmark, doubly synchronised trophozoite-stage parasites were exposed to increasing concentrations of TCMDC-135051 (0.25–4 µM) for 60 min to identify a concentration that did not adversely affect parasite growth (Fig. 1A).

In these experiments, parasites exposed to TCMDC-135051 at concentrations between 0.25 and 1 µM for 60 min showed no significant morphological effects on trophozoite progression through the parasite life cycle. The parasites matured into schizonts and produced invasive daughter merozoites. These parasites were maintained in culture, and no deleterious effects on development, maturation, or reinvasion of fresh erythrocytes were observed (Fig. 1B and C).

At higher concentrations (2 and 4 μM), however, trophozoites-stage parasites showed significant morphological changes and appeared visibly shrunken at 96 h post invasion (hpi) (Fig. 1B). This corresponds to the observed decreased parasitaemia (Fig. 1C). Based on this evidence, exposure of trophozoite-stage parasites to 1 μM TCMDC-135051 inhibitor for 60 min was chosen as the suitable condition to investigate the effects of *Pf*CLK3 inhibition on gene transcription and RNA splicing. For parasite treatment, sampling, and RNA preparation, see supplementary figure, Fig. S1.

### Inhibition of *Pf*CLK3 results in disruption of essential gene transcription

**Fig 2:**
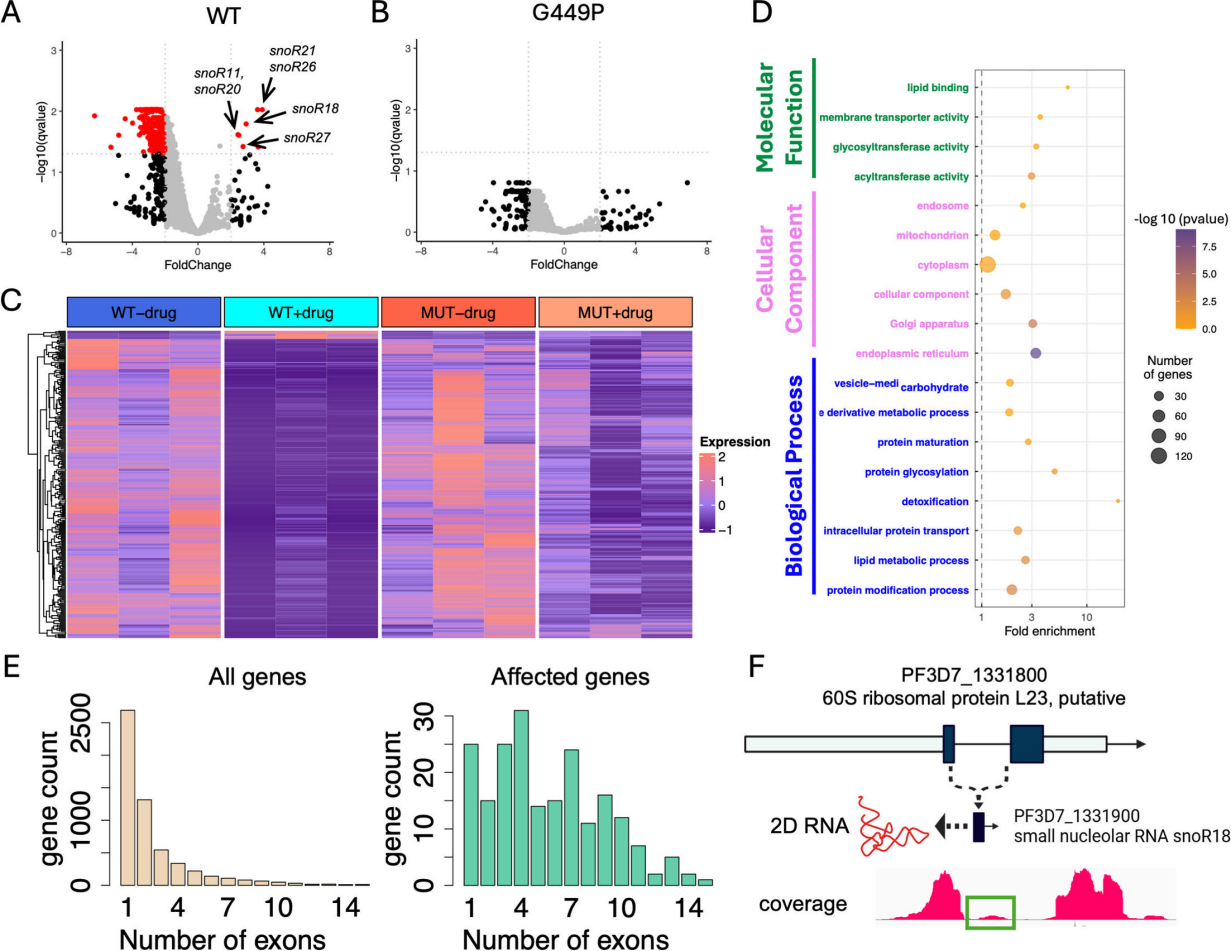
Gene expression changes in response to PFCLK3 inhibition. (**A and B**) Volcano plots showing differential gene expression in WT (**A**) and G449P (8) parasites under TCMDC-135051 treatment. Red = genes that reached the threshold of differential expression (log2 fold change > 2 and corrected *P*-value < 0.05, Table S1A; (**C**) Heatmap of 214 genes differentially expressed in response to PfCLK3 inhibition in WT parasites, showing their expression in both WT anid G449P samples. WT-drug = wild type untreated, WT + drug = wild type treated, MUT­drug = G449P untreated, MUT + drug = G449P treated. Each row on the *x*-axis represents an individual gene, and each column represents one biological replicate. (**D**) GO term enrichment in differentially expressed genes. Only terms with *P*-value of one-sided Fisher's exact test < 0.05 are shown (Table S1B). (**E**) Distribution of genes according to number of exons in whole transcriptome (left) and amongst differentially expressed genes (right), and (**F**) the production of small nucleolar RNA (snoR18) from the intron of 60S ribosomal protein and its coverage in one of the samples.

The inhibition of *Pf*CLK3 impacted gene transcription, resulting in the downregulation of 218 transcripts and upregulation of 7 in wild type (WT) 3D7 parasites (Fig. 2A; Table S1). In contrast, treatment of the TCMDC-135051 resistant mutant parasite strain, G449P, exhibited a markedly reduced transcriptional response to treatment (Fig. 2B and C; Table S1). Importantly, under control conditions (i.e., without drug exposure), there were no detectable differences in gene transcription between WT and G449P mutant parasites. This indicates that the G449P mutation alone has no effect on transcription under no drug conditions (Fig. S3A). Overall, these findings strongly support the conclusion that TCMDC-135051 specifically targets *Pf*CLK3 to modulate gene transcription.

The downregulated genes represent a subsection of previous microarray data (generated following exposure to the same inhibitor under lethal conditions and, thus, containing multiple secondary changes) (Fig. S3B). Ontology analysis of downregulated genes upon *Pf*CLK3 inhibition revealed enrichment in pathways critical to multiple essential parasite processes. These included genes involved in protein translation, intracellular trafficking, and post-translational modification as well as genes regulating mitochondrial function and metabolic processes (Fig. 2D), including Folate Transporter 1, ERAD-associated E3 ubiquitin-protein ligase HRD1, and *DP-diacylglycerol--inositol 3-phosphatidyltransferase* (Fig. S4) (19–21). The most striking feature of these downregulated genes, however, is that 92.5% contain introns. Since approximately 50% of *P. falciparum* genes are intronic (22), this is a significant over-representation (*P* = 2.2E-16, Fisher’s exact test) of intron-containing genes (Fig. 2E).

Only seven transcripts are upregulated in response to treatment. Notably, six of these encode small nucleolar RNA (snoRNA) species involved in RNA processing and modification, generated through intronic processing of larger host genes—typically those encoding ribosomal proteins (Fig. 2F; Fig. S5). In mammalian cells, the snoRNA HBII-52 binds splicing silencers to regulate efficient splicing, and that loss of this interaction is a hallmark of transcript-processing-deficient diseases like Prader-Willi Syndrome (23). While snoRNAs have not been extensively investigated in *Plasmodium*, the observed upregulation may inhibit splicing silencers to promote efficient splicing following the inhibition of the key splicing partner, *Pf*CLK3. Alternatively, the observed accumulation of snoRNAs may reflect aberrant splicing events in the host genes, in which dysfunctional processing of primary transcripts due to *Pf*CLK3 inhibition generates hybrid mRNA–snoRNA species. Such defective splicing would lead to intron retention and the consequent accumulation of intronic regions containing snoRNA fragments, a phenomenon that has been reported previously in systems with compromised spliceosome function (24). Together, these data prompted a more direct examination of the impact of *Pf*CLK3 inhibition on RNA splicing.

### Effects of *Pf*CLK3 inhibition on RNA splicing

**Fig 3:**
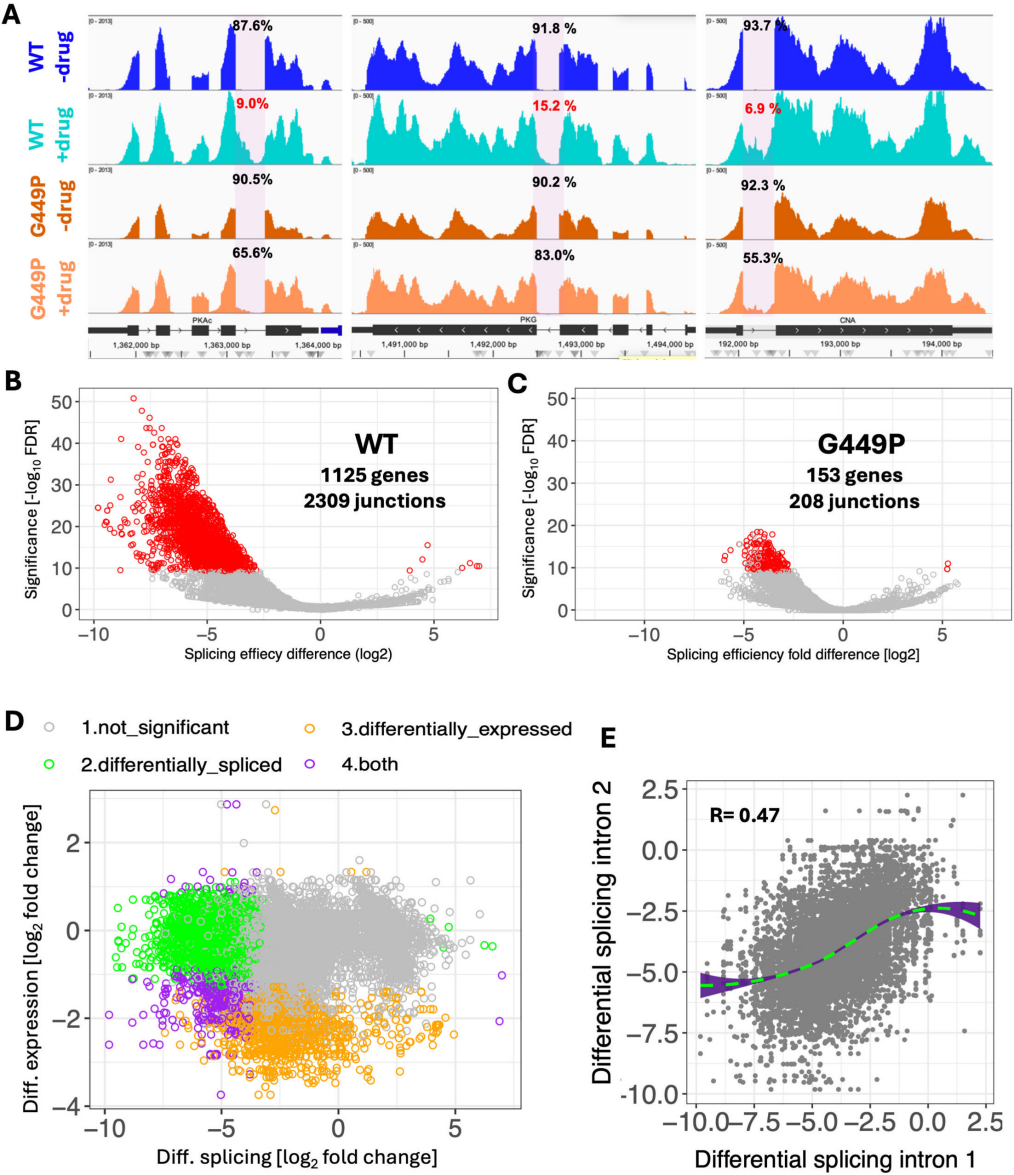
Analysis on global splicing landscape under the influence ofTCMDC-135051 treatment. (**A**) Coverage plots showing disturbed intron processing in WT and G449P samples. As shown, splicing of intrans was efficiently completed in untreated WT in the range of about 90% compared to treated samples at between 7% and 15% for the same regions for PKAc, PKG, and CNA genes, reducing splicing by more than 85%. This demonstrates the strong role of PfCLK3 in splicing. However, in G449P parasites, inhibition reduces splicing only by a maximum of 37% for PKAc, PKG, and CNA. For each condition, only 1 of the 3 replicates is shown. (**B and C**) Volcano plots showing differential splicing in WT and G449P parasites upon treatment with TCMDC-135051. Each point represents a spicing junction. Significantly differentially spliced genes (>25% in splicing difference and FDR value <0.001 ). (**D**) Comparison between differential splicing and differential expression. Fold difference in splicing efficiency in WT parasites after TCMDC-135051 pulse vs fold difference in the expression of the gene in which the junction is located. Points colored according to the FDR values for both differential splicing and expression. (**E**) Effect of TCMDC-135051 on junctions within the same transcript. Differential splicing was analyzed between each intron pair located within the same transcript. Trend line represents a local polynomial regression model predicting differential splicing of intron 2 based on intron 1. R = Pearson correlation coefficient.

A visual inspection of coverage across several genes revealed that, while clear intron boundaries were present in untreated parasites, splicing in treated WT samples was significantly disrupted, with multiple reads spanning introns. In untreated WT samples, approximately 90% of each transcript appeared to be correctly processed, while in treated WT samples, proper splicing dropped to as low as 10% for some junctions (Fig. 3A). In contrast, G449P parasites showed little to no defects in differential gene splicing between treated and untreated conditions for the same transcripts.

Global analysis of splicing efficiency identified 2,309 junctions across 1,125 genes that were significantly affected by the action of the compound and *Pf*CLK3 inhibition (Fig. 3B and C, Table S2). Further investigation of the nature of this defect revealed little correlation between gene expression level and splicing defect (Fig. S6A and B), thereby ruling out potential data analysis artifacts. While many mis-spliced genes were differentially expressed (*n* = 163), the two phenomena were largely independent, with many genes either correctly spliced but differentially expressed or mis-spliced without changes in expression (Fig. 3D). As for differential expression, the G499P mutant was largely unaffected with only 208 junctions across 153 genes compared to WT, confirming the leading role of *Pf*CLK3 in this phenomenon (Fig. 3C).

**Fig 4:**
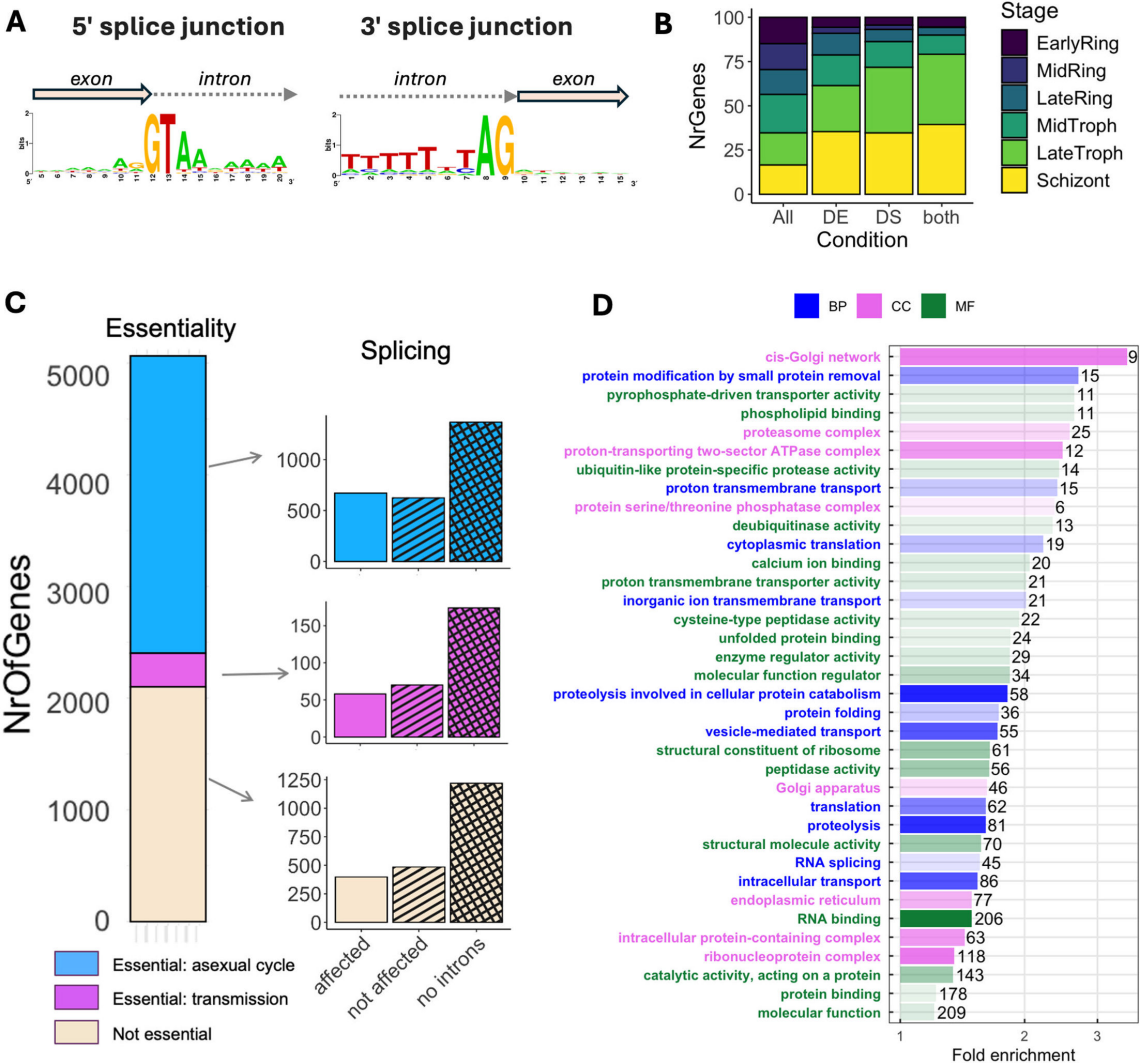
Analysis of junctions misplaced upon CLK3 inhibition in schizonts: (**A**) Affected junctions’ base composition at the 5′ and 3′ end, (**B**) timing of expression of differentially expressed and differentially spliced genes according to Painter et al. (25). All = all transcripts in the genome, DE = differentially expressed, DS = differentially spliced, both = differentially spliced and differentially expressed. (**C**) Essentiality status of affected genes (using Zhang et al. (3) for asexual replication and combination of Russell et al. (26) and Stanway et al. (27) for sexual development). (**D**) GO terms highly enriched (corrected *P*-value < 0.05) in differentially spliced gene subset. The terms are colored by category: BP = Biological Process, CC = Cellular Component, MF= Molecular Function. The color intensity is reverse proportional to the *P*-value of one-sided Fisher's exact test. Number of genes with affected introns is shown next to the fold enrichment bar. Full list of affected processes and genes contributing to them can found in Table S3.

Affected junctions were further analysed to assess the selectivity of drug action, given that the majority of the 10,566 detected introns showed no evidence of drug-induced changes. The fold-change comparison of junction pairs within the same transcript showed a moderate positive correlation (*r* = 0.47; Fig. 3E); however, the magnitude of impact sometimes varied substantially among introns. All disturbed junctions were canonical major spliceosome junctions (of the GU/AG type) with no enrichment of any motif in either the 200 bp flanking regions or the introns themselves (Fig. 4A). Expression profile analysis shows a disproportionate representation of genes peaking in the trophozoite and schizont stages (25), consistent with the timing of the drug pulse delivery (Fig. 4B). However, multiple genes with stage-specific expression in other life stages were also detected (e.g., Thioredoxin 1 [PfTrx1]; secreted ookinete protein, putative [PSOP24]; protein phosphatase PPM2; AP2 domain transcription factor AP2-O5, putative [AP2-O5]), suggesting that the drug may induce similar defects in gametocytes or ring stages. Taken together, these findings indicate that TCMDC-135051 efficacy against *Pf*CLK3 does not correlate with sequence-specific binding or with expression timing but may instead depend on other factors such as RNA transcript turnover or translation kinetics.

The implications of splicing inhibition for the parasite biology were significant. GO terms analysis revealed 36 significantly enriched terms (FDR < 0.05 after Benjamini Hochberg correction) (Fig. 4D; Table S3). These involved some of the most essential processes for parasite survival such as translation (GO:0006412), RNA splicing (GO:0008380), and binding (GO:0003723) as well as proteolysis (GO:0006508) and protein folding (GO:0006457). This pattern potentially reflects increased turnover of the RNA- and protein-processing machinery in response to widespread splicing defects which paradoxically results in genes in these pathways to be more prone to aberrant processing, leading to even greater dysregulation.

The analysis of the essentiality status of affected genes according to references (3, 5, 28) showed that at least 576 were essential for asexual replication, and 54 genes were essential for later stages such as gametocytogenesis or mosquito transmission (26, 27) (Fig. 4C). This suggests that splicing inhibition would lead to both a reduction of asexual parasitaemia and decreased malaria transmission.

### PCR confirms inhibition of splicing in selected transcripts

**Fig 5:**
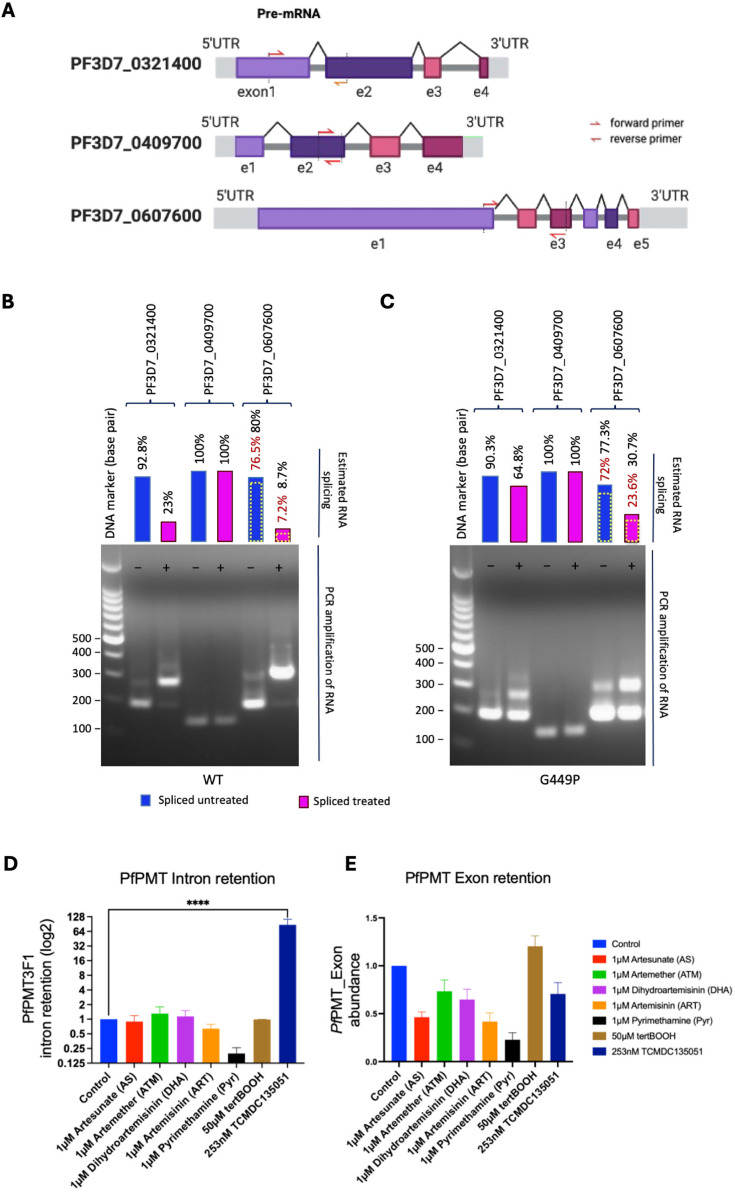
Validation of the effect for individual junctions. (**A**) Schematic diagram of selected genes with disrupted splicing in WT parasites when treated with the PfCLK3 inhibitor. Oligonucleotide PCR primers designed flanking selected predicted single intrans that might be alternatively spliced as in PF3D7 _0321400; an exonic region that would not be spliced, as in PD3D7 _0409700; or spanning across three exons (flanking two intrans) as shown with the gene PD3D7 _0607600 (Created with BioRender.com). (**B** and **C**) PCR amplification of target regions showing splicing levels in the absence/presence of inhibitor in WT and G449P parasites. Top panel shows splicing completing as detected from the RNA­seq data confirming alternative splicing in untreated (−, blue bar) and TCMDC135051 treated (+, pink bar) WT and G449P parasites. While splicing efficiency overlap between WT and G449P in untreated samples, there are obvious difference in splicing comparing treated WT and G449P parasites. (**D**) Comparing intron retention between TCMDC-135051 treated WT parasites to other commonly artemisinin and its derivates. Higher intron retention in TCMDC-135051 confirms splicing inhibition. (Error bars represent standard error of three independent experiments in triplicates.) (**E**) Exon retention was evaluated, but no significant difference was observed.

To validate the splicing disruption observed from the bioinformatic analysis, we selected genes with introns that were dysregulated in the presence of the *Pf*CLK3 inhibitor. We then investigated whether these disrupted splicing events could be detected by PCR amplification in both WT and G449P parasites. Primers were designed to amplify both spliced and unprocessed transcripts (Fig. 5A), ensuring the detection of splicing defects involving a single intron (PF3D7_0321400) or multiple introns (PF3D7_0607600).

In the absence of the inhibitor, splicing efficiency was comparable between WT and G449P parasites (e.g., 76.5% and 80%, respectively). However, in the presence of *Pf*CLK3 inhibitor, splicing efficiency in WT parasites was drastically reduced to 7.2% and 8.7% for first and second junctions, respectively. In contrast, G449P parasites maintained high splicing efficiencies of 23.6% and 30.7% at the same junctions—representing a threefold better splicing efficiency. These results further indicate that the differential splicing observed is directly attributable to the role *Pf*CLK3 in regulating splicing (Fig. 5B and C).

Since splicing efficiency is not typically assessed in standard drug assays, we employ a qPCR assay to examine the behavior of one of the affected spliced junctions under a range of commonly used sub-lethal antimalarial drug treatments. These included the current first-line therapy components—artemisinin and its derivatives used in combination therapy (artesunate, artemether, and dihydroartemisinin)—as well as pyrimethamine, tertBOOT, and TCMDC-135051. Intron retention was quantified following parasite exposure to each drug (Fig. 5D). As expected, parasites treated with the *Pf*CLK3 selective inhibitor TCMDC-135051 exhibited significantly higher intron retention, consistent with splicing disruption. In contrast, exon expression remained unchanged across all treatments (Fig. 5E).

## DISCUSSION

The *Plasmodium* genome is highly compact, characterised by a dense arrangement of open reading frames (ORFs) and frequently overlapping untranslated regions (UTRs) of neighboring genes. Despite this compactness, approximately half of the genes in *Plasmodium falciparum* (52.5%) contain introns—often multiple—making the parasite highly reliant on an efficient splicing machinery to sustain essential cellular functions. The presence of introns also allows the production of multiple isoforms from a single transcript via alternative splicing—something that has been shown for several *Plasmodium* genes. While the functional roles of most of these isoforms have yet to be characterised, the parasite likely employs alternative splicing to diversify its proteome, supporting precise regulation of gene expression across distinct cell types, developmental stages, and host environments. As an example, alternative splicing is important for determining gametocyte sexes (male for female gametocytes) after commitment (8, 10).

The study presented here demonstrates that *Pf*CLK3-inhibition leads to widespread splicing dysregulation, affecting approximately 20% of the transcriptome and 22% of all junctions. At prolonged high compound concentration, this disruption likely results in large accumulation of unprocessed transcripts and proteins, ultimately causing parasite death, thus explaining the mechanism of action of CLK3 inhibitors. However, in our experimental set-up, we purposely used a concentration that had a minimal immediate impact on parasite progression through the life cycle. Most parasites were able to successfully complete the generation of progeny merozoites and subsequent invasion. This suggests that parasites may persist for a period using pre-accumulated proteins or transcripts, or by producing excessive amounts of RNA, allowing them to survive even when only a small fraction is correctly processed. It also implies that, in contrast to higher eukaryotic cells, *Plasmodium* may tolerate high proportion (>90%) of aberrant (nonsense) transcripts of some genes without immediate damage or functional consequences. This interpretation would be consistent with the fact that not many of these transcripts are actively degraded (as they show no global decrease in differential expression analysis) as well as the fact that nonsense-mediated transcript decay is not essential for *Plasmodium* (29). These findings should be considered during further optimisation of *Pf*CLK3 inhibitors, including decisions regarding dosing regimens, partner drugs, and treatment duration. They also indicate that *Pf*CLK3 inhibitors could serve as valuable tools for studying mRNA and protein turnover dynamics across different parasite life cycle stages—an area that remains poorly understood.

Detailed analysis of the affected spliced junctions revealed that, although numerous genes are affected by *Pf*CLK3 inhibition, the effect is not global. Many transcripts continue to be processed correctly while others remained completely unprocessed. The fact that affected transcripts are expressed mainly in schizonts suggest that the effect may be influenced by RNA turnover (i.e., only transcripts that are highly expressed and degraded within the 1-h window of the drug action are affected). However, even within the same transcript, processing efficiency varies according to different junctions (Fig. 3A and E), suggesting that some splicing events are more sensitive to *Pf*CLK3 inhibition than others. This suggests that *Pf*CLK3 activity is not uniformly required across all splicing events but instead appears to be particularly critical for specific classes of introns or transcript contexts. This is consistent with previous work performed on the *Pf*CLK3 homolog in yeast, Prp4, where its inhibition led to decreased splicing of only a subset of introns, characterised by “weak” 5′ splice sites or suboptimal branch point sequences (14). Similarly, in *Toxoplasma gondii*, the inhibition of the CLK3 homolog, *Tg*PRP4, led to disturbed splicing of the second intron of many genes (30). The mechanistic basis of CLK3 selectivity at these junctions requires further investigation across different parasite life stages and under varying drug-pulse intervals. Identifying particularly sensitive junctions would deepen our understanding of *Plasmodium* biology and could enable the development of new assays, facilitating medium-throughput screening for additional splicing inhibitors.

In addition to the findings described above, which advanced our understanding of *Plasmodium* biology, this work further validates *Pf*CLK3 as a potential drug target for the development of new antimalarials. To address the growing resistance to current front-line malaria treatments, next generation anti-malarial drugs must act on novel targets and possess unique mechanism of action. Ideally, these compounds should be effective across multiple stages of the parasite life cycle—including liver stage (providing prophylactic activity), the asexual blood stages (for curative treatment), and the sexual (gametocyte) stages to block transmission (31, 32). The molecular mechanism of action of TCMDC-135051 supports the potential of *Pf*CLK3 inhibitors to meet these criteria.

Although the development of highly selective protein kinase inhibitors is inherently challenging, the availability of a high-resolution X-ray crystal structure of *Pf*CLK3 in complex with TCMDC-135051 (6) has enabled a rational structure-based drug design approach. Application of this strategy within a medical chemistry program has yielded highly selective *Pf*CLK3 inhibitors. The molecular effect of *Pf*CLK3 inhibition, as described here, together with previous studies demonstrating activity against clinical isolates with confirmed resistance background, multistage killing of asexual parasites, cross-species activity, transmission-blocking effects, and liver stage-activity against sporozoites (4, 17), supports the notion that inhibition of *Pf*CLK3 could deliver a radical cure. Therefore, *Pf*CLK3 inhibitors are currently being developed to meet the target candidate profiles (TCP) defined by Medicines for Malaria Venture (MMV), specifically for a cure (TCP-1) and chemoprevention (TCP-2) (32).

## MATERIALS AND METHODS

### Parasite culture and drug assays

Asexual blood stage *P. falciparum* 3D7 parasites were cultured at 37°C in RPMI (Roswell Park Memorial Institute) media supplemented with 0.5% Albumax II (ThermoFischer Scientific) as a serum substitute, 0.292 g L-glutamine, 0.05 g hypoxanthine, 2.3 g sodium bicarbonate, 0.025 g gentamicin, 5.957 g HEPES, and 4 g dextrose in 1 litee RPMI. Parasites were maintained at 4% haematocrit using O+ erythrocytes from anonymous male donors, provided by the UK Blood and Transfusion service. Cultures were maintained in a controlled gas atmosphere (90% Nitrogen, 5% Carbon dioxide, 5% oxygen) (33). Parasitaemia was routinely monitored by Giemsa staining of methanol-fixed air-dried thin blood smears, visualised by light microscopy. Parasite cultures were synchronised by isolating mature schizont-stage parasites on a cushion of 60% Percoll (GE Healthcare). Purified schizonts were incubated in complete media at 37°C with fresh erythrocytes for 1–4 h in a shaking incubator to allow new merozoites to invade the erythrocytes. Remaining schizonts were removed by a second Percoll purification, yielding tightly synchronised ring-stage parasites.

### Establishment of drug concentrations, RNA sample collection and preparation

To determine the optimal sublethal concentration of TCMDC-135051 that would allow assessment of its effect on parasite pre-mRNA processing and splicing without killing the parasites, doubly synchronized ring-stage parasites (0–3 h post-invasion [hpi])—WT and G449P—at 1% parasitaemia and 4% haematocrit (HCT) were aliquoted into a 96-well flat-bottom plate and cultured up to the late trophozoite stage (30 hpi). The parasites were then exposed to varying concentrations of TCMDC-135051 (0, 0.25, 0.5, 1, 2, and 4 μM) for 1 h at 37°C. Drug pressure was removed by washing the cultures three times with complete media and parasites incubated further up to 120 hpi. Giemsa-stained thin smears were collected at 30- and 48 hpi and every 24 h up to 120 hpi to monitor parasite replication (Fig. 1A through C). As proxy for parasitaemia, Sybr green assay was used to measure DNA staining as direct measure for parasitaemia. Assessment of parasite morphology and parasitaemia indicates that up to 1 μM drug exposure had little effect on parasite replication and was used in subsequent steps.

To collect samples for RNA sequencing, doubly synchronised WT and G449P parasites were cultured for 30 hpi (±2 h) and then treated with 1 μM TCMDC-135051 inhibitor or vehicle (DMSO) for 1 h at 37°C. Parasitised erythrocytes were harvested by centrifugation and stored in TRIzol in −80°C until RNA extraction. Parasite total RNA was extracted using the Qiagen RNeasy Mini Kit according to the manufacturer’s instructions. RNA quantity and quality were measured by NonoDrop 2000 (Thermo Fisher Scientific). Both treatment and controls were run in triplicates under the same culture conditions. Complementary DNA, cDNA was generated using QuantiTect Reverse Transcription Kit according to manufacturer’s instruction.

### RNA-sequencing data generation

Purified RNA quality was checked using Agilent Bioanalyser and Nanodrop spectrophotometer. Illumina TruSEQ RNA library prep and sequencing reagents were used. The samples initially went through a round of Poly-T selection followed by cDNA synthesis using random primers. The samples were paired-end multiplex sequenced (2 × 150 base pairs) on the illumina HiSeq 2500 platform, and at least 30 million reads were generated for each sample. The quality of the raw sequencing reads was assessed using FastQC software.

### Data analysis

#### Pre-processing

The RNA-seq reads (FASTQ) was mapped against *P. falciparum* reference genome (downloaded from PlasmoDB v. 56) using splice-aware aligner hisat2 (v. 2.2.1) (34). Resulting BAM files were sorted, indexed, and filtered for the mapping quality (only reads with MAPQ >10 were kept) using Samtools suite (v. 1.17) (35) and visually inspected using IGV genome browser (36).

### Differential gene expression analysis

The number of reads mapping against each of the annotated genes was calculated with *featureCounts* function from subread software package (v.2.0.0) using the genome feature file (gff) corresponding to the used genome version. R software with edgeR function package was used to normalise the data, estimate dispersions, and fit generalised linear model to the data set with default parameters, as specified in the software vignette. Pairwise contrasts were set up between the two lines in the same conditions (G449P vs WT) or between one line in two different conditions using r quasi-likelihood (QLF)-test. A log2-fold change cut off in gene expression of >2 and *P*-values of statistical test with Bonferoni multiple testing correction of <0.05 were used to call differentially expressed genes.

### Differential splicing analysis

Conventional tools used for splicing analysis based on isoform usage quantification proved suboptimal for the *Plasmodium* genome, likely due to its high gene density and uneven exon coverage. Similar issue has been discussed previously (10). To address this, we developed a custom approach method to quantify differences in intron processing across parasite lines and treatment conditions (Fig. S2). This approach was centered on individual intron processing quantification. Within each gene, we considered all the candidate splice junctions, i.e., all the junctions in the reference annotation from PlasmoDB, plus all the genomic intervals flanked by the canonical splice sites GT/AG. For each splice junctions in each RNA-Seq library, we quantified two types of reads: those supporting splicing—i.e. reads split by Hisat2 aligner (34) to bridge the two adjacent exons—and those supporting intron retention—i.e. reads partially overlapping an exon and the adjacent intron. This quantification was performed using featureCounts (37) and custom scripts (see below). Differential splicing is the difference in the proportion of spliced vs unspliced reads in one condition vs another condition. To assess this difference, we applied the method developed for differential methylation (38) since effectively methylated vs unmethylated counts at a cytosine are equivalent to spliced vs unspliced counts at a splice junction. In brief, the method applies the generalised linear model (GLM) strategy implemented in edgeR package (21) where the vector counts (spliced and unspliced) at a junction is modeled as a GLM with negative binomial distribution with covariates being the treatment effect and the count type (spliced or unspliced). Differential splicing is then the interaction effect between treatment and count type. To reduce statistical and biological noise, we tested only junctions accumulating at least 15 reads in all the libraries from at least 1 condition. This workflow is available as a Snakemake pipeline at https://github.com/glaParaBio/denovo-detection-of-differential-splicing. While this approach has some limitations inherent to short reads processing (in particular, it does not allow to analyse the full transcript but focusses on individual junctions), this approach outperformed alternatives that report differential splicing and expression separately. To confirm this, several junctions were validated by manual inspection of coverage plots (e.g., Fig. 4A) and by PCR amplification (Fig. 5B). Importantly, the same algorithm has shown no differential splicing between the untreated lines, showing lack of false positives in the analysis pipeline further validating the algorithm.

For each pairwise comparison, a *DGEList* object was created comprising the counts from relevant libraries, along with sample information and library sizes, which were calculated by summing the splice and unspliced reads counts for each sample. The design matrix was generated according to reference (38) and used for dispersion estimation. A negative binomial generalized log-linear model was fitted to the read counts of each intron, and modified likelihood ratio test (LRT) was conducted using either the strain or drug treatment as a contrast condition. The resulting table containing log-fold changes of the proportion of reads spliced, and *P*-values with Bonferroni correction (FDR) were generated and used to filter the differentially spliced introns. Introns with >25% changes in splicing proportions between the conditions and *q* values <0.001 were considered as differentially spliced/retained. Visual inspection of selected junctions was performed by generating *.bw files using deeptools v. 3.3.2 and viewing them with Integrative Genomics Viewer v2.3.91.

### GO terms analysis and comparison with existing data sets

GO terms analysis was carried out using topGO package in R and GO term annotations downloaded from https://plasmodb.org (39). Terms with an enrichment test of *P* value < 0.05 were considered significant and are included in the Table S3.

The number of exons per gene and its analysis were calculated based on gff file and plotted using custom R script.

To compare gene transcription timing with the effect of the compound on splicing, data sets describing *de novo* transcription at each stage of the parasite asexual life cycle (25) were downloaded. A chi-square test was then performed to assess enrichment of genes whose peak *de novo* transcription occurred at each given stage.

To access the effect on genes essential for different life stages, results from essentiality screens in asexual (3), gametocyte (26), and mosquito stages (27) were downloaded. Where data sets were generated from species than other *P. falciparum*, genes were converted by orthology. Each data set was then filtered for essential genes (MIS score ≤0.5 for asexual stage screen, phenotype label “loss of function” for gametocyte screen, and status label “reduced” for mosquito stages). Mosquito and gametocyte-essential genes were pooled and classified as “transmission essential.” The number of affected and unaffected genes in each category was calculated. Genes lacking introns but showing excision rates >10% detected in any of the data sets were classified as “no intron genes.”

### PCR amplification of cDNA

To show splicing disruption using standard PCR, selected genes with introns were chosen. PCR primers were designed on exon regions flanking intron sites (Fig. 5; Table S4). Amplifications of parasite cDNA were done using standard methods, and the PCR product ran on 1% agarose gel to visualise amplified products.

## Data Availability

The pipeline used for the differential splicing analysis is available at https://github.com/glaParaBio/denovo-detection-of-differential-splicing. Illumina sequencing data have been deposited in the NCBI Gene Expression Omnibus (GEO) under the accession number GSE278703. For the purpose of open access, the author(s) have applied a Creative Commons Attribution (CC BY) licence to any Author Accepted Manuscript version arising from this submission.

## SUPPLEMENTAL MATERIAL

Fig. S1 aac.01561-25-s0001.tiff Schematic workflow for enriching parasites, RNA purification, and sequencing and data analysis.

Fig. S2 aac.01561-25-s0002.tiff Strategy for identifying differentially spliced junctions in Plasmodium.

Fig. S3 aac.01561-25-s0003.tiff Differential expression between WT and G449P mutant; Comparison of differentially expressed genes win our dataset (orange) with the previous microarray data investigations (grey).

Fig. S4 aac.01561-25-s0004.tiff Examples of coverage of three genes differentially expressed in the response to drug treatment.

Fig. S5 aac.01561-25-s0005.tiff Schematic representation of snoRNAs affected by TCMDC-135051 treatment: (A-F) position of 6 out of 7 overexpressed snoRNA within the "host" genes.

Fig. S6 aac.01561-25-s0006.tiff Differential splicing analysis across different conditions.

Table S1 aac.01561-25-s0007.xlsx Differential expression in response to TCMDC135051.

Table S2 aac.01561-25-s0008.xlsx Differential splicing in response to TCMDC135051.

Table S3 aac.01561-25-s0009.xlsx GOterms affected by splicing.

Table S4 aac.01561-25-s0010.xlsx List of a selection of genes with introns.

ASM does not own the copyrights to Supplemental Material that may be linked to, or accessed through, an article. The authors have granted ASM a non-exclusive, world-wide license to publish the Supplemental Material files. Please contact the corresponding author directly for reuse.

## References

[1] Worl. 2025. World Health Organization

[2] Solyakov L, Halbert J, Alam MM, Semblat J-P, Dorin-Semblat D, Reininger L, Bottrill AR, Mistry S, Abdi A, Fennell C, Holland Z, Demarta C, Bouza Y, Sicard A, Nivez M-P, Eschenlauer S, Lama T, Thomas DC, Sharma P, Agarwal S, Kern S, Pradel G, Graciotti M, Tobin AB, Doerig C. 2011. Global kinomic and phospho-proteomic analyses of the human malaria parasite Plasmodium falciparum. Nat Commun 2:565. doi:10.1038/ncomms1558 22127061

[3] Zhang M, Wang C, Otto TD, Oberstaller J, Liao X, Adapa SR, Udenze K, Bronner IF, Casandra D, Mayho M, Brown J, Li S, Swanson J, Rayner JC, Jiang RHY, Adams JH. 2018. Uncovering the essential genes of the human malaria parasite Plasmodium falciparum by saturation mutagenesis. Science 360:eaap7847. doi:10.1126/science.aap7847 29724925 PMC6360947

[4] Alam MM, Sanchez-Azqueta A, Janha O, Flannery EL, Mahindra A, Mapesa K, Char AB, Sriranganadane D, Brancucci NMB, Antonova-Koch Y, et al.. 2019. Validation of the protein kinase PfCLK3 as a multistage cross-species malarial drug target. Science 365:eaau1682. doi:10.1126/science.aau1682 31467193

[5] Oberstaller J, Xu S, Naskar D, Zhang M, Wang C, Gibbons J, Pires CV, Mayho M, Otto TD, Rayner JC, Adams JH. 2025. Supersaturation mutagenesis reveals adaptive rewiring of essential genes among malaria parasites. Science 387:eadq7347. doi:10.1126/science.adq7347 39913589 PMC12131478

[6] Brettell SB, Janha O, Begen A, Cann G, Sharma S, Olaniyan N, Yelland T, Hole AJ, Alam B, Mayville E, Gillespie R, Capper M, Fidock DA, Milligan G, Clarke DJ, Tobin AB, Jamieson AG. 2024. Targeting PfCLK3 with covalent inhibitors: a novel strategy for malaria treatment. J Med Chem 67:18895–18910. doi:10.1021/acs.jmedchem.4c01300 39441986 PMC11571108

[7] Kern S, Agarwal S, Huber K, Gehring AP, Strödke B, Wirth CC, Brügl T, Abodo LO, Dandekar T, Doerig C, Fischer R, Tobin AB, Alam MM, Bracher F, Pradel G. 2014. Inhibition of the SR protein-phosphorylating CLK kinases of Plasmodium falciparum impairs blood stage replication and malaria transmission. PLoS One 9:e105732. doi:10.1371/journal.pone.0105732 25188378 PMC4154858

[8] Eshar S, Altenhofen L, Rabner A, Ross P, Fastman Y, Mandel-Gutfreund Y, Karni R, Llinás M, Dzikowski R. 2015. PfSR1 controls alternative splicing and steady-state RNA levels in Plasmodium falciparum through preferential recognition of specific RNA motifs. Mol Microbiol 96:1283–1297. doi:10.1111/mmi.13007 25807998

[9] Chen M, Manley JL. 2009. Mechanisms of alternative splicing regulation: insights from molecular and genomics approaches. Nat Rev Mol Cell Biol 10:741–754. doi:10.1038/nrm2777 19773805 PMC2958924

[10] Yeoh LM, Goodman CD, Mollard V, McHugh E, Lee VV, Sturm A, Cozijnsen A, McFadden GI, Ralph SA. 2019. Alternative splicing is required for stage differentiation in malaria parasites. Genome Biol 20:151. doi:10.1186/s13059-019-1756-6 31370870 PMC6669979

[11] Ltzelberger M, F N. 2012. The Prp4 kinase: its substrates, function and regulation in pre-mRNA splicing. *In* Protein phosphorylation in human health

[12] Schneider M, Hsiao HH, Will CL, Giet R, Urlaub H, Lührmann R. 2010. Human PRP4 kinase is required for stable tri-snRNP association during spliceosomal B complex formation. Nat Struct Mol Biol 17:216–221. doi:10.1038/nsmb.1718 20118938

[13] Dellaire G, Makarov EM, Cowger JeffJM, Longman D, Sutherland HGE, Lührmann R, Torchia J, Bickmore WA. 2002. Mammalian PRP4 Kinase Copurifies and Interacts with Components of Both the U5 snRNP and the N-CoR Deacetylase Complexes. Mol Cell Biol 22:5141–5156. doi:10.1128/MCB.22.14.5141-5156.2002 12077342 PMC139773

[14] Eckert D, Andrée N, Razanau A, Zock-Emmenthal S, Lützelberger M, Plath S, Schmidt H, Guerra-Moreno A, Cozzuto L, Ayté J, Käufer NF. 2016. Prp4 kinase grants the license to splice: control of weak splice sites during spliceosome activation. PLoS Genet 12:e1005768. doi:10.1371/journal.pgen.1005768 26730850 PMC4701394

[15] Brettell SB, Fuentes-Guerra Bustos C, Sharma S, Cann G, Carruthers LV, Begen A, Milligan G, Clarke DJ, Tobin AB, Jamieson AG. 2026. Shaping antimalarials: a geometry-first approach to PfCLK3 covalent inhibitors. J Med Chem 69:3378–3395. doi:10.1021/acs.jmedchem.5c03342 41618901 PMC12910650

[16] Brettell SB, Cann G, Begen A, Sharma S, Mahindra A, Carruthers LV, Milligan G, Clarke DJ, Tobin AB, Jamieson AG. 2025. Lysine targeting covalent inhibitors of malarial kinase PfCLK3. RSC Med Chem 16:3530–3540. doi:10.1039/d5md00335k 40521344 PMC12164071

[17] Mahindra A, Janha O, Mapesa K, Sanchez-Azqueta A, Alam MM, Amambua-Ngwa A, Nwakanma DC, Tobin AB, Jamieson AG. 2020. Development of potent PfCLK3 inhibitors based on TCMDC-135051 as a new class of antimalarials. J Med Chem 63:9300–9315. doi:10.1021/acs.jmedchem.0c00451 32787140 PMC7497403

[18] Sorber K, Dimon MT, Derisi JL. 2011. RNA-Seq analysis of splicing in Plasmodium falciparum uncovers new splice junctions, alternative splicing and splicing of antisense transcripts. Nucleic Acids Res 39:3820–3835. doi:10.1093/nar/gkq1223 21245033 PMC3089446

[19] Wengelnik K, Vial HJ. 2007. Characterisation of the phosphatidylinositol synthase gene of Plasmodium species. Res Microbiol 158:51–59. doi:10.1016/j.resmic.2006.11.005 17223316

[20] Chung DWD, Ponts N, Prudhomme J, Rodrigues EM, Le Roch KG. 2012. Characterization of the ubiquitylating components of the human malaria parasite’s protein degradation pathway. PLoS One 7:e43477. doi:10.1371/journal.pone.0043477 22912882 PMC3422240

[21] Salcedo-Sora JE, Ochong E, Beveridge S, Johnson D, Nzila A, Biagini GA, Stocks PA, O’Neill PM, Krishna S, Bray PG, Ward SA. 2011. The molecular basis of folate salvage in Plasmodium falciparum: characterization of two folate transporters. J Biol Chem 286:44659–44668. doi:10.1074/jbc.M111.286054 21998306 PMC3247980

[22] Gardner MJ, Hall N, Fung E, White O, Berriman M, Hyman RW, Carlton JM, Pain A, Nelson KE, Bowman S, et al.. 2002. Genome sequence of the human malaria parasite Plasmodium falciparum. Nature 419:498–511. doi:10.1038/nature01097 12368864 PMC3836256

[23] Bruce S, Stewart DJ. 2006. Regulation of alternative splicing by snoRNAs, p 574 . Cold Spring Harbor Laboratory Press.

[24] Liu Y, DeMario S, He K, Gibbs MR, Barr KW, Chanfreau GF. 2022. Splicing inactivation generates hybrid mRNA-snoRNA transcripts targeted by cytoplasmic RNA decay. Proc Natl Acad Sci USA 119. doi:10.1073/pnas.2202473119 PMC9351541 35878033

[25] Painter HJ, Chung NC, Sebastian A, Albert I, Storey JD, Llinás M. 2018. Genome-wide real-time in vivo transcriptional dynamics during Plasmodium falciparum blood-stage development. Nat Commun 9:2656. doi:10.1038/s41467-018-04966-3 29985403 PMC6037754

[26] Russell AJC, Sanderson T, Bushell E, Talman AM, Anar B, Girling G, Hunziker M, Kent RS, Martin JS, Metcalf T, Montandon R, Pandey V, Pardo M, Roberts AB, Sayers C, Schwach F, Choudhary JS, Rayner JC, Voet T, Modrzynska KK, Waters AP, Lawniczak MKN, Billker O. 2023. Regulators of male and female sexual development are critical for the transmission of a malaria parasite. Cell Host Microbe 31:305–319. doi:10.1016/j.chom.2022.12.011 36634679 PMC7616090

[27] Stanway RR, Bushell E, Chiappino-Pepe A, Roques M, Sanderson T, Franke-Fayard B, Caldelari R, Golomingi M, Nyonda M, Pandey V, Schwach F, Chevalley S, Ramesar J, Metcalf T, Herd C, Burda P-C, Rayner JC, Soldati-Favre D, Janse CJ, Hatzimanikatis V, Billker O, Heussler VT. 2019. Genome-scale identification of essential metabolic processes for targeting the plasmodium liver stage. Cell 179:1112–1128. doi:10.1016/j.cell.2019.10.030 31730853 PMC6904910

[28] Bushell E, Gomes AR, Sanderson T, Anar B, Girling G, Herd C, Metcalf T, Modrzynska K, Schwach F, Martin RE, Mather MW, McFadden GI, Parts L, Rutledge GG, Vaidya AB, Wengelnik K, Rayner JC, Billker O. 2017. Functional profiling of a plasmodium genome reveals an abundance of essential genes. Cell 170:260–272. doi:10.1016/j.cell.2017.06.030 28708996 PMC5509546

[29] McHugh E, Bulloch MS, Batinovic S, Patrick CJ, Sarna DK, Ralph SA. 2023. Nonsense-mediated decay machinery in Plasmodium falciparum is inefficient and non-essential. mSphere 8:e0023323. doi:10.1128/msphere.00233-23 37366629 PMC10449492

[30] Swale C, Bellini V, Bowler MW, Flore N, Brenier-Pinchart M-P, Cannella D, Belmudes L, Mas C, Couté Y, Laurent F, Scherf A, Bougdour A, Hakimi M-A. 2022. Altiratinib blocks Toxoplasma gondii and Plasmodium falciparum development by selectively targeting a spliceosome kinase. Sci Transl Med 14:eabn3231. doi:10.1126/scitranslmed.abn3231 35921477

[31] Belete TM. 2020. Recent progress in the development of new antimalarial drugs with novel targets. Drug Des Devel Ther 14:3875–3889. doi:10.2147/DDDT.S265602 PMC7519860 33061294

[32] Siqueira-Neto JL, Wicht KJ, Chibale K, Burrows JN, Fidock DA, Winzeler EA. 2023. Antimalarial drug discovery: progress and approaches. Nat Rev Drug Discov 22:807–826. doi:10.1038/s41573-023-00772-9 37652975 PMC10543600

[33] Amambua-Ngwa A, Button-Simons KA, Li X, Kumar S, Brenneman KV, Ferrari M, Checkley LA, Haile MT, Shoue DA, McDew-White M, et al.. 2023. Chloroquine resistance evolution in Plasmodium falciparum is mediated by the putative amino acid transporter AAT1. Nat Microbiol 8:1213–1226. doi:10.1038/s41564-023-01377-z 37169919 PMC10322710

[34] Kim D, Langmead B, Salzberg SL. 2015. HISAT: a fast spliced aligner with low memory requirements. Nat Methods 12:357–360. doi:10.1038/nmeth.3317 25751142 PMC4655817

[35] Li H, Handsaker B, Wysoker A, Fennell T, Ruan J, Homer N, Marth G, Abecasis G, Durbin R, 1000 Genome Project Data Processing Subgroup. 2009. The sequence Alignment/Map format and SAMtools. Bioinformatics 25:2078–2079. doi:10.1093/bioinformatics/btp352 19505943 PMC2723002

[36] Robinson JT, Thorvaldsdóttir H, Winckler W, Guttman M, Lander ES, Getz G, Mesirov JP. 2011. Integrative genomics viewer. Nat Biotechnol 29:24–26. doi:10.1038/nbt.1754 21221095 PMC3346182

[37] Liao Y, Smyth GK, Shi W. 2014. FeatureCounts: an efficient general purpose program for assigning sequence reads to genomic features. Bioinformatics 30:923–930. doi:10.1093/bioinformatics/btt656 24227677

[38] Chen Y, Pal B, Visvader JE, Smyth GK. 2017. Differential methylation analysis of reduced representation bisulfite sequencing experiments using edgeR. F1000Res 6:2055. doi:10.12688/f1000research.13196.1 29333247 PMC5747346

[39] Aurrecoechea C, Brestelli J, Brunk BP, Dommer J, Fischer S, Gajria B, et al.. 2009. PlasmoDB: a functional genomic database for malaria parasites. Nucleic Acids Res 37:D539–D543. doi:10.1093/nar/gkn814 18957442 PMC2686598

